# An inducible transposon mutagenesis approach for the intracellular human pathogen *Chlamydia trachomatis*

**DOI:** 10.12688/wellcomeopenres.16068.1

**Published:** 2021-11-16

**Authors:** Colette E. O'Neill, Rachel J. Skilton, Jade Forster, David W. Cleary, Sarah A. Pearson, David J. Lampe, Nicholas R. Thomson, Ian N. Clarke

**Affiliations:** 1 Clinical and Experimental Sciences, University of Southampton, Southampton, Hampshire, SO166YD, UK; 2 Cancer Sciences, University of Southampton, Southampton, SO16 6YD, UK; 3 Department of Biological Sciences, Duquesne University, Pittsburgh, Pennsylvania, 15116, USA; 4 Bacterial Genomics and Evolution, Wellcome Trust Sanger Institute, Hinxton, Cambridgeshire, CB10 1SA, UK

**Keywords:** Chlamydia trachomatis, MinION, transposon mutagenesis, inducible promoter, anhydrotetracycline, theophylline, riboswitch, dual-control regulon, TraDIS, genetics

## Abstract

**Background:** *Chlamydia trachomatis* is a prolific human pathogen that can cause serious long-term conditions if left untreated. Recent developments in *Chlamydia* genetics have opened the door to conducting targeted and random mutagenesis experiments to identify gene function. In the present study, an inducible transposon mutagenesis approach was developed for *C. trachomatis* using a self-replicating vector to deliver the transposon-transposase cassette - a significant step towards our ultimate aim of achieving saturation mutagenesis of the *Chlamydia* genome.

**Methods:** The low transformation efficiency of *C. trachomatis* necessitated the design of a self-replicating vector carrying the transposon mutagenesis cassette (i.e. the Himar-1 transposon containing the beta lactamase gene as well as a hyperactive transposase gene under inducible control of the *tet* promoter system with the addition of a riboswitch). *Chlamydia* transformed with this vector (pSW2-RiboA-C9Q) were induced at 24 hours post-infection. Through dual control of transcription and translation, basal expression of transposase was tightly regulated to stabilise the plasmid prior to transposition.

**Results:** Here we present the preliminary sequencing results of transposon mutant pools of both *C. trachomatis* biovars, using two plasmid-free representatives: urogenital strain *C. trachomatis* SWFP- and the lymphogranuloma venereum isolate L2(25667R). DNA sequencing libraries were generated and analysed using Oxford Nanopore Technologies’ MinION technology. This enabled ‘proof of concept’ for the methods as an initial low-throughput screen of mutant libraries; the next step is to employ high throughput sequencing to assess saturation mutagenesis.

**Conclusions:** This significant advance provides an efficient method for assaying *C. trachomatis* gene function and will enable the identification of the essential gene set of *C. trachomatis*. In the long-term, the methods described herein will add to the growing knowledge of chlamydial infection biology leading to the discovery of novel drug or vaccine targets.

## Introduction

*Chlamydia trachomatis* is a common human pathogen that infects epithelial cells of mucosal sites where it causes distinct clinical manifestations including blinding trachoma and the sexually transmitted infection chlamydia. *C. trachomatis* has a small chromosome, at around 1.04Mb ^1^, as many biochemical pathways became redundant and lost or truncated through genome reduction during adaptation/evolution in the intracellular environment. The *C. trachomatis* genome is densely packed with protein coding sequences, but less than half of the *c.* 900 predicted genes have been assigned a function - most of which were identified through homology to genes in other species rather than empirical evidence from *C. trachomatis* itself ^2^. This is because the field of chlamydial genetics is relatively new, with the first reproducible genetic transformation system developed in 2011, nearly seventy years after bacterial transformation was first described ^3^. Since the transformation barrier was breached, important strides have been taken (as recently reviewed ^4^). However, a key question that remains is which genes are essential to chlamydial survival? The answer will give insight into the principle cellular processes that govern *Chlamydia* biology. To progress, this approach needs the technology of saturation mutagenesis.

Two main methods of gene function analysis currently exist in *Chlamydia* research: targeted mutagenesis ^5, 6^ and chemical mutagenesis ^7, 8^. Whilst effective for identifying gene function on a gene-by-gene basis, these cannot assay for genome-wide gene essentiality due to the laborious and time-consuming methods required. Choosing genes for such a mutagenic approach without knowledge of essentiality is a high risk, inefficient and wasteful venture. In other bacterial systems, essential gene sets are identified using saturation transposon mutagenesis. Various protocols exist, including TraDIS ^9^, Tn-seq ^10^ and INSeq ^11^, all of which utilize random transposon insertion followed by very high-throughput sequencing to assay gene function across the whole genome.

In this study we devised a TraDIS-based approach for use in *C. trachomatis*, offering a novel approach for saturation mutagenesis and thus identifying gene essentiality/functionality. TraDIS (Transposon Directed Insertion Site sequencing) was first developed for use in *Salmonella* Typhi to identify the genes that are involved in bile tolerance ^9^. To date, twenty further bacterial species have been subjected to TraDIS, including human pathogens such as *Escherichia coli* ^12^, *Staphylococcus aureus* ^13^, *Yersinia pestis* ^14^, *Bordetella pertussis* ^15^, *Streptococcus pyogenes* ^16^ and *Mycobacterium tuberculosis* ^17^. However, the TraDIS approach has yet to be applied to any obligate intracellular bacterial species.

Most free-living bacterial species can be simply transformed with transposon DNA that has been pre-treated with transposase, enabling immediate transposition in the bacterial cell upon transformation; commercial kits based on this technology are readily available for such systems ^18, 19^. However, this option is not possible for those working with intracellular organisms such as *Chlamydia* as the transformation efficiency is prohibitively low. Rather, the transposon needs to be delivered to the bacterial cell accompanied by the transposase gene so that transposition can occur *in situ*. This was recently achieved in *Chlamydia* through the construction and isolation of individual transposon mutants, showing proof-of-principle that transposons are active in *C. trachomatis* ^20^ and the closely related species, *Chlamydia muridarum* ^21^. The authors used a non-replicating vector carrying the *Himar1* mariner transposon and the gene encoding the transposase enzyme (under control of a constitutive chlamydial promoter) to cause immediate mutation of chlamydia upon its transformation, after which the plasmid was lost. Due to low transformation efficiency, this method resulted in very few insertions per experiment but enabled individual mutant selection. However, to achieve our aim of saturation transposon mutagenesis, it is necessary to introduce the transposon mutagenesis components on a plasmid that replicates in *Chlamydia* so that transformants can first be expanded in cell culture, followed by induction of transposition once a high concentration of transformants is obtained. This will enable insertional mutagenesis at scale.

Through use of a dual-control regulon incorporating both transcriptional and translational regulation, we successfully introduced the *Himar1* transposon and a partial reversion mutant of the C9 hyperactive transposase ^22^ to *C. trachomatis* SWFP- and L2(25667R) on a single self-replicating *Chlamydia* shuttle vector (pSW2-RiboA-C9Q). This vector is stable over many passages, enabling us to achieve high infectivity before induction of transposition. Herein, we describe the first application of a TraDIS-based approach to *C. trachomatis* (or any obligate intracellular bacterium) that will enable saturation transposon mutagenesis of the *C. trachomatis* genome. This will enable the identification of the minimal essential genome, knowledge that can be used to help determine gene function and identify potential novel targets for drug development.

## Methods

### Ethics statement

All genetic manipulations and containment work were approved under the UK Health and Safety Executive Genetically Modified Organisms (contained use) regulations notification number GM57, 10.1 entitled “Genetic transformation of Chlamydiae”.

### Bacteria and eukaryotic cell culture

*E. coli* strain DH5α was used for the construction of plasmid vectors in this study ( Table 1). *E. coli* strain C2925 is a Dam-/Dcm- strain used to produce unmethylated vector DNA prior to transformation into *Chlamydia* ^23^. *Chlamydia trachomatis* SWFP- and L2(25667R) are naturally-occurring plasmid-free clinical isolates ^24, 25^ obtained from the Chlamydia Biobank.

**Table 1.** Bacteria and plasmids used in this study.

	Name	Features	Reference/Source
***E. coli***	DH5α	Basic *E. coli* host for cloning and maintenance of plasmid vectors.	Hanahan *et al.*, 1985 ^26^ Grant *et al.*, 1990 ^27^. Supplied by ThermoFisher.
	C2925	Methyltransferase deficient (dam-/dcm-) chemically competent *E. coli*	Supplied by New England Biolabs (C2925I)
***C. trachomatis***	SWFP-	Plasmid-free urogenital strain, serovar F.	Wang *et al.*, 2013 ^24^. Supplied by the Chlamydia Biobank (CT107)
	L2(25667R)	Plasmid-free LGV strain L2	Peterson *et al.*, 1990 ^25^. Supplied by the Chlamydia Biobank (CT109)
**Plasmids**	p2TK2-SW2-mCh	Chlamydia shuttle vector carrying Spectinomycin resistance marker aadA2 and mCherry (red fluorescent protein).	Cortina *et al.*, 2019 ^28^. Provided by Prof Isabelle Derre (University of Virginia, USA)
	pSW2-RiboA-C9	As for p2TK2-SW2-mCh but also carrying the *Himar1* transposon and C9 hyperactive transposase under control of the *tet* promoter and riboswitch A	This study
	pSW2-RiboA-C9Q	As for pSW2-RiboA-C9 but with amino acid substitution R131Q	This study

***E. coli culture*.** *E. coli* was routinely grown in Luria Bertani (LB) broth or agar (1%). When required, ampicillin was used at 50µgml ^-1^ and spectinomycin at 100µgml ^-1^. Broth cultures were grown 16 hours with agitation (200 rpm) at 37°C. Solid cultures were grown overnight at 37°C.

***C. trachomatis culture*.** *C. trachomatis* was grown in mouse McCoy fibroblast monolayers (ECACC, catalogue number 90010305) using DMEM (Gibco) medium (with GlutaMax, without Pyruvate) supplemented with 10% foetal calf serum (Gibco). Cycloheximide was added at 1µgml ^-1^ and when needed penicillin was added at 10Uml ^-1^ or spectinomycin at 500µgml ^-1^ to select for transformants. Cells were infected as previously described ^29^ and incubated at 37°C / 5% CO _2_ for 44–48 hours, or until mature inclusions were observed. Chlamydiae were harvested as previously described ^30^.

### Vector construction

**Figure 1.**
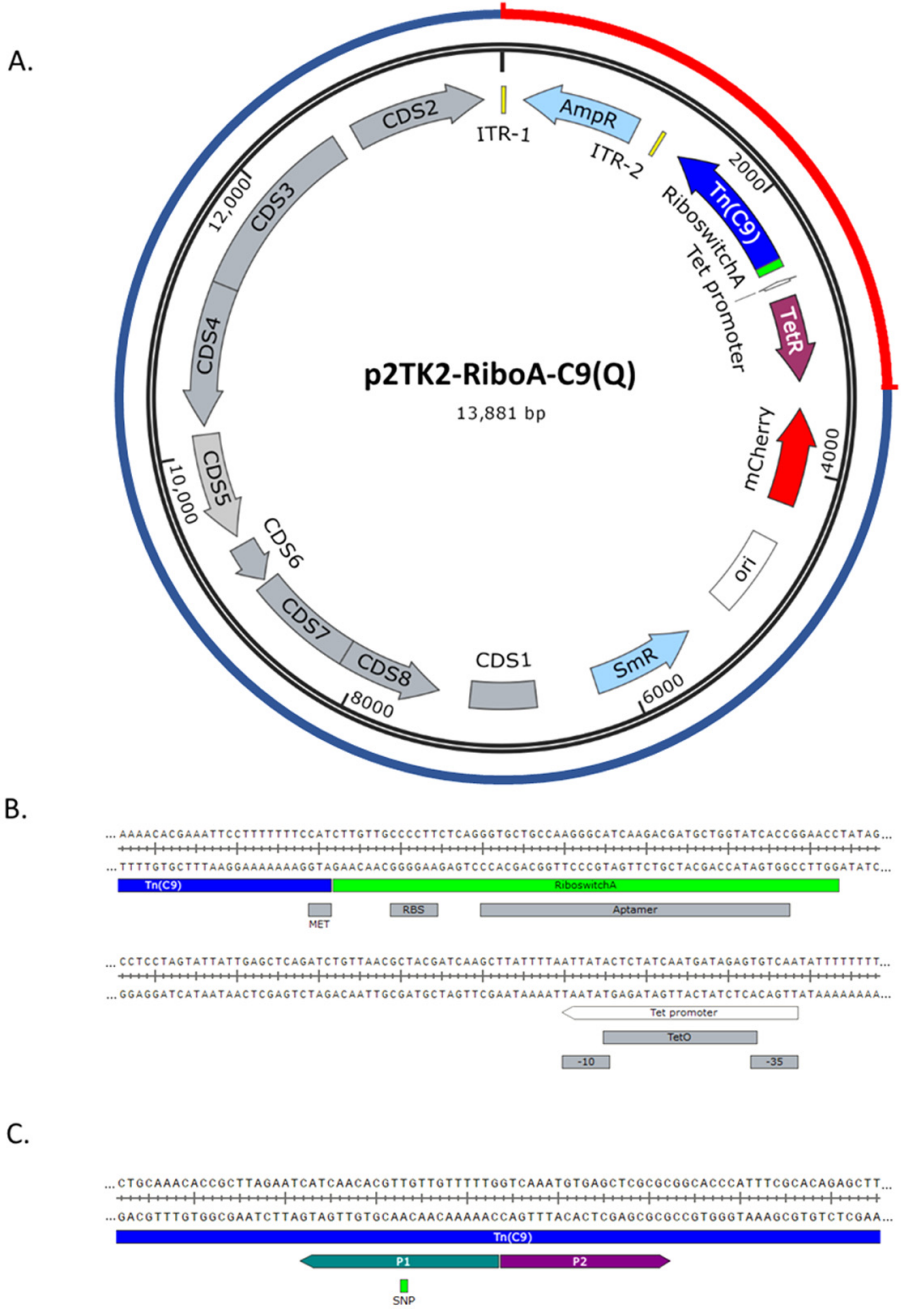
The self-replicating *C. trachomatis* transposon delivery vector. **A**. Vector map of pSW2-RiboA-C9 and pSW2-RiboA-C9Q. The outer circle represents p2TK2-RiboA-C9 vector origins: the blue portion indicates the shuttle vector pSW2-mCh-C9; the red portion is the synthesised transposon-transposase cassette. ITR1 and ITR2 are the inverted terminal repeats either side of the ampicillin resistance gene (AmpR), forming the transposon unit. Tn(C9) is the hyperactive C9 transposon (with or without the single nucleotide polymorphism conferring the R131Q substitution). The *tet* promoter and riboswitch A are indicated. *TetR* is the tetracycline repressor gene; *mCherry* is the red fluorescent reporter; ori is the pUC origin of replication for *E. coli* and *SmR* is the spectinomycin resistance gene. The grey CDS features represent genes derived from the *C. trachomatis* SW2 plasmid. **B**. The riboswitch A sequence (green) and its features shown in context with the start codon (MET) of the tranpsposase gene (blue) and the upstream *tet* promoter (white). **C**. The position of the single nucleotide polymorphism (SNP) in the C9 transposase gene conferring the amino acid change R131Q, introduced by divergent PCR of the vector with primers P1 and P2, yielding vector pSW2-RiboA-C9Q.

***pSW2-RiboA-C9*.** As basal transcription of the transposase appeared to be responsible for the inability to transform chlamydia with pSW2-mCh-C9 ^30^, we investigated the coupling of transcriptional and translational regulation by the addition of a theophylline-dependent riboswitch between the *tet* promoter and C9 transposase start codon ^31, 32^ ( Figure 1b). Riboswitch variant A was selected as this was found to be the most repressed in *E. coli*, with only a very low expression level even in the presence of theophylline ^31^. The transposon-transposase cassette was commercially synthesised (Eurogentec, Belgium), incorporating *MmeI* restriction digest sites into the inverted terminal repeat (ITR) sequences for future optimisation of sequencing protocols ^33^. The cassette was provided in the pUC57-kan vector, which confers kanamycin resistance in addition to the ampicillin resistance carried by the transposon. Upon receipt, the DNA was immediately transformed into *E. coli* DH5α as described previously ^30^. Colonies were sub-cultured into 5ml of LB broth/ampicillin (50µgml ^-1^) and plasmid DNA was prepared using a SmartPure Plasmid kit (Catalogue number SK-PLPU-100, Eurogentec, Belgium). The transposon-transposase cassette was excised from the cloning vector by digestion with *SalI* restriction enzyme, then ligated into the unique *SalI* site in shuttle vector p2TK2-SW2-mCh yielding vector pSW2-RiboA-C9 ( Figure 1a); clones were then selected with ampicillin (50ugml ^-1^). Plasmid DNA from overnight liquid cultures of DH5α/pSW2-RiboA-C9 was prepared using the SmartPure Plasmid Kit (Eurogentec, Belgium), and transformed into Dam-/Dcm- *E. coli* strain C2925 using ampicillin selection. After clonal purification, C2925/pSW2-RiboA-C9 DNA was purified from overnight liquid culture. The sequence of pSW2-RiboA-C9 was confirmed by commercial sequencing (CCIB DNA Core, Massachusetts, USA).

***pSW2-RiboA-C9Q*.** Vector pSW2-RiboA-C9Q was constructed from pSW2-RiboA-C9 using divergent PCR amplification of the whole vector with Primer_1 (CAAAAACAAC **A** ACGTGTTGATG) and Primer_2 (GTCAAATGTGAGCTCGCGC). Primer_1 contains the base change from G to A (shown in bold and underlined) to result in amino acid substitution R to Q at amino acid position 131 ( Figure 1c). PCR products were analysed on a 1% agarose gel and cleaned using the SmartPure Gel kit (Eurogentec). 10U of *DpnI* was added to the cleaned PCR product (20µl) and 5µl of Cutsmart 10x buffer (NEB). The volume was adjusted to 50µl with nuclease-free water and incubated at 37°C for one hour and heat inactivated at 80°C for 20 minutes. Next, this total volume was subjected to phosphorylation using 10U of T4 Polynucleotide Kinase, with 10x T4 Ligase Buffer (Promega), adjusted to 25µl with nuclease-free water. This was incubated at 37°C for 30 minutes, then inactivated at 65°C for 20 minutes. 10U of T4 DNA ligase was then added, and the reaction was incubated overnight at 16°C. The next day the whole ligation was mixed with 100µl of chemically competent *E. coli* DH5a, incubated on ice for 20 minutes then heat-shocked at 42°C for 50 seconds. 1ml of LB medium was added to the mixture and incubated at 37°C on an orbital shaker for one hour. Cells were then pelleted at 4000 × *g* for five minutes, resuspended in 50µl of LB, then spread onto LB agar plates containing spectinomycin (100µgml ^–1^) and ampicillin (50µgml ^–1^). These were incubated overnight at 37°C; colonies were picked and sub-cultured into 10ml LB/amp/spec, incubated overnight at 37°C on an orbital shaker and plasmid DNA was isolated using the SmartPure plasmid kit. The sequence of pSW2-RiboA-C9Q was confirmed by whole plasmid sequencing at MGH CCIB DNA Core (Massachusetts General Hospital, USA).

### Transformation of *C. trachomatis*

Transformations of *C. trachomatis* SWFP- with pSW2-RiboA-C9 and pSW2-RiboA-C9Q were performed as described by Cortina *et al.* ^28^; p2TK2-SW2-mCh was used a transformation control. Transformants were selected with spectinomycin at 500µgml ^-1^ immediately after transformation (T0) and throughout the following passages. *Chlamydia* were passaged until a high infectivity was obtained for each of the transformants.

### Induction of transposon mutagenesis

To produce mutant pools for sequencing, McCoy cell monolayers grown overnight in 25cm ^2^ tissue culture flasks were infected with *C. trachomatis* SWFP-/pSW2-RiboA-C9Q or L2(25667R)/pSW2-RiboA-C9Q at a multiplicity of infection of 1 (MOI=1), as previously described ^29^. At 24 hours post infection, transposition was induced by adding 10ngml ^-1^ anhydrotetracycline (ATc) and 2mM theophylline to the flask in the existing cell culture medium. A non-induced control was included for comparison. *Chlamydia* were incubated for a further 20–24 hours (until mature inclusions were observed) at 37°C/5% CO _2_, after which they were harvested and stored at -70°C as previously described ^29^.

### Protein expression analysis

To optimise expression timings and inducer concentrations, Western blot immunodetection was performed as previously described ^29^. Briefly, *C. trachomatis* SWFP- carrying plasmid pSW2-RiboA-C9Q was grown in mouse McCoy cells in 6-well trays for 24 hours at 37°C/5%CO _2_. After 24 hours, ATc was added at 20ngml ^-1^, along with 0, 2 or 10mM theophylline (additional control wells without ATc contained theophylline at these same concentrations). Following induction, the infections were incubated for a further 24 hours before being harvested. The infected monolayer was detached from each well by scraping with a 1ml pipette tip. The entire volume was transferred to a 1.5ml microcentrifuge tube and pelleted at 14,000 × *g* for two minutes. The pellet was resuspended in 1× sample loading buffer containing β-mercaptoethanol, boiled for five minutes and subjected to SDS polyacrylamide gel electrophoresis. The separated proteins were then blotted onto polyvinylidene diflouride (PVDF) Immobilon membrane (EMD Millipore) in Pierce Fast Semi-Dry Buffer (ThermoFisher Scientific) using a Pierce Fast Semi-Dry Blotter. The membrane was blocked in a solution of PBS/0.05% Tween-20 (PBS-T) and 10% skimmed milk for one hour at room temperature, then a 1:5000 dilution of primary mouse polyclonal antisera to C9 transposase ^30^ was added in 1% milk/PBS-T for one hour at RT. Following three washes with PBS-T, the membrane was transferred to PBS-T/1% milk containing horseradish peroxidase-conjugated goat- anti-mouse antibody as per manufacturer’s instructions (BioRad). The membrane was washed three more times, then incubated with Pierce ECL Western Blotting Substrate (Thermo Scientific) before exposure to Kodak BioMax XAR film.

### Mutant passaging experiment

As a step towards essential gene identification, induced cultures of *C. trachomatis* (the input libraries) were passaged and the surviving population was harvested (output library), with the aim of identifying surviving mutants. *C. trachomatis* SWFP- transformed with p2TK2-SW2-C9Q was grown in McCoy cells in 25cm ^2^ tissue culture flasks and induced as described above (section 2.5). At the end of the developmental cycle, the *Chlamydia* were harvested and passaged onto overnight McCoy cell monolayers, and incubated at 37°C/5% CO _2_. At the end of the developmental cycle, the surviving population was harvested, as previously described ^30^.

### Genomic DNA extraction

***Extraction for proof of principle experiments*.** For library preparation and subsequent sequencing, 100µl (10%) of harvested induced transposon mutant pools of SWFP- and L2(25667R) was pelleted by centrifugation at 14,000 × *g* for two minutes and DNA extracted using the Macherey-Nagel™ NucleoSpin™ Tissue kit (Fisher Scientific 11982262), following the manufacturer’s instructions.

***Extraction for identification of surviving mutants in output library*.** After harvesting the input and output libraries from the passaging experiment (as described above), 300µl (30% of the total harvest) of infected cellular material (containing both residual host cell debris and chlamydia EBs and RBs) was retained for DNA extraction. Harvests were pelleted by centrifugation at 14,000 × *g* for two minutes and DNA extracted using the Nucleospin Tissue Kit (Machery Nagel), following the manufacturer’s instructions.

### Library preparation and sequencing

The MinION sequencing library was prepared from neat DNA extracts, following the Rapid Sequencing protocol (SQK-RAD004, ONT). 400ng of DNA was adjusted to 7.5µl with nuclease-free water, and 2.5µl of fragmentation mix (FRA) was added. This was incubated at 30°C for one minute and 80°C for one minute. Directly to this, without prior DNA clean-up, 1µl of Rapid Adapter (RAP) was added, and the library was incubated at room temperature for five minutes before being loaded onto a FLO-MIN106 flow-cell (ONT). All flow cells had been used previously in an unrelated project (not chlamydial DNA) and were cleaned prior to use with the Flow Cell Wash Kit (ONT); the flow cell QC scan at the start of the run identified between 300–800 pores available for sequencing in all sequencing runs. Sequencing by MinION Mk1b proceeded for 48–72 hours (without barcoding as only a single sample was sequenced), until most sequencing pores were inactive. MinKNOW version 20.06.5 was used to run the MinION and perform basecalling. 

### Sequence analysis and identification of transposon insertion locations

As the MinION generates long-read sequencing data, it was not possible to use the TraDIS data analysis methodology (TraDIS Toolkit ^34^) to analyse this dataset; instead tools available on the free online bioinformatics platform Galaxy were used to run all analyses unless otherwise stated. Initial visualization and processing of long-read sequencing data was performed using Nanoplot (v.1.28.2) ^35^.

Sub-sets of fastQ files containing all the read data (<4000 fastQ reads per file) were combined into sets of fastQ files using the Collapse Collection tool. Each compiled fastQ file was then converted to a fastA file using the “Fastq to Fasta” tool. Next, makeblastdb (with default parameters) was used to make a BLAST database of the reads in a fastA reads file, then megablast was used to search for the transposon sequence among the dataset. The output table was downloaded and the perl script “fastagrabber.pl” ^36^ was used to extract the whole read for each match to the transposon. The output fastA file containing these reads was loaded back into Galaxy, and Medaka Consensus Pipeline was used to map all reads containing a transposon sequence to the chromosome sequence of SWFP- or L2(25667R) (this step removed any reads that originated from the transposon-delivery vector). The output BAM (and indexing BAI) files were downloaded and mapped onto reference SWFP- (ENA accession: HE605380) or L2(25667R) (HE601954) chromosomal sequences using the Artemis genome browser (v18.1.0), which enabled the manual identification of each insert location. SnapGene (v5.2.1) was used to view each transposon-containing read that mapped to the chromosome; the position of the transposon within the read was identified, then the flanking sequences were manually mapped to the SWFP- or L2(25667R) chromosome map to identify the exact location of the transposon insertion. This process was repeated for each of the collections of fastQ reads.

## Results

### Transformation of *C. trachomatis* with the pSW2-RiboA-C9 delivery vector was successful, but transformants failed to expand under spectinomycin selection in cell culture

**Figure 2.**
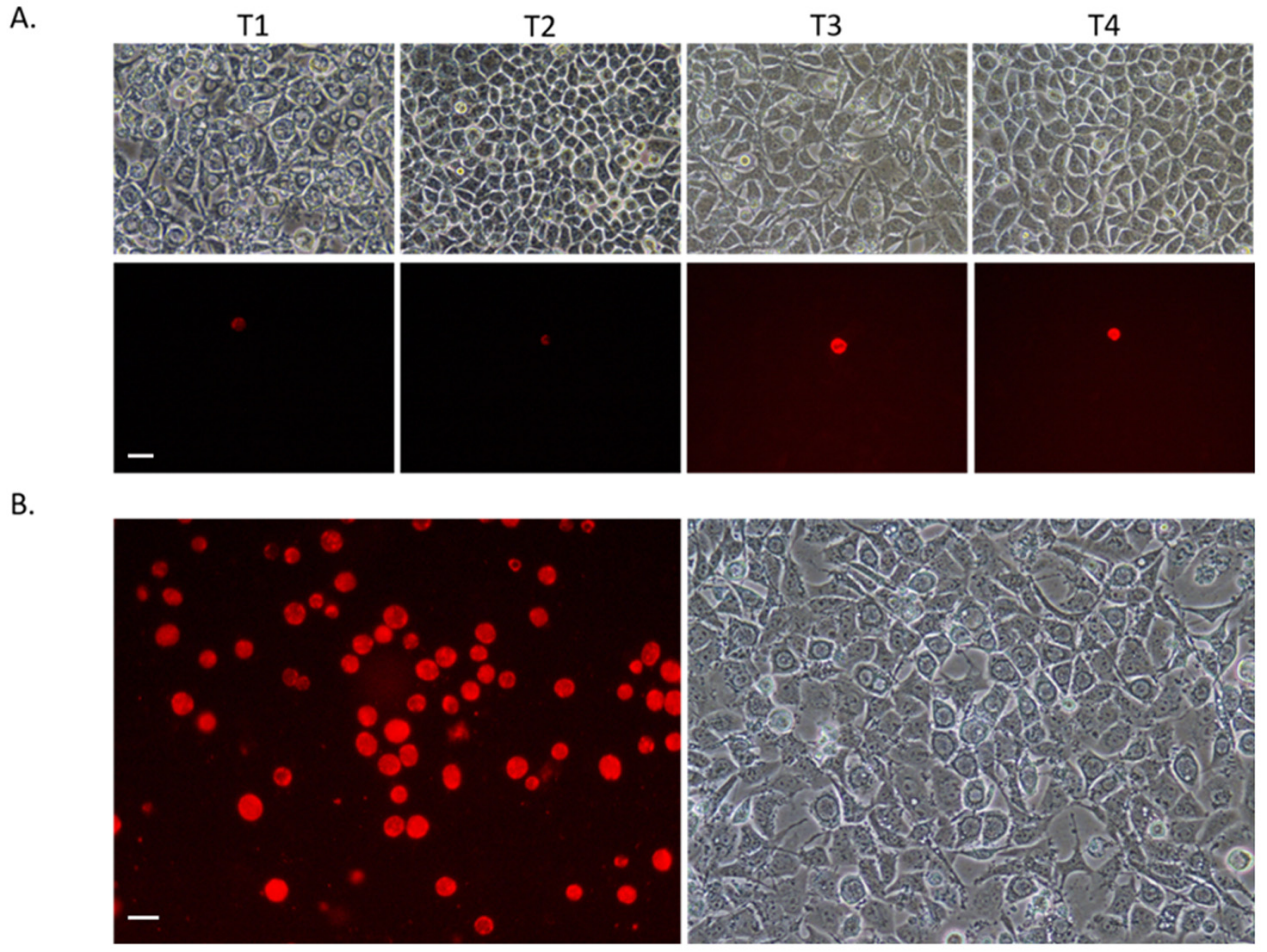
Photomicrographs under UV light (with red filter) and phase contrast. *Chlamydia* transformants generated in this study, grown in mouse McCoy fibroblasts. Scale bars represent 20µM. **A**. Serial passage of SWFP-/pSW2-RiboA-C9. The whole harvest was passaged at each stage, but the frequency of transformants did not expand. T1-T4 are representative passage numbers. At T1, most cells contain inclusions with typical aberrant body phenotypes consistent with non-transformed *Chlamydia* grown under penicillin selection. By T2 these non-transformed Chlamydia were no longer present consistent with their penicillin-sensitive phenotype and by passage 6, the transformants were also lost. **B**. SWFP- transformed with pSW2-RiboA-C9Q. Successful expansion in cell culture; representative image from passage 6 is shown.

Previous attempts to introduce the transposon and transposase to *C. trachomatis* on a single replicating vector were unsuccessful; we hypothesised that basal expression from the *tet* promoter was causing premature transposition, resulting in immediate vector loss and hence killing of early transformants ^30^. To reduce this basal expression, we investigated the use of translational regulation by incorporating a riboswitch between the promoter and start codon of the C9 transposase. We chose riboswitch A as a starting point; in *E. coli* (and most other bacterial species tested) riboswitch A results in the lowest-level expression in its induced and un-induced states ^31^; this was chosen to counteract the high activity of the C9 transposase and give the best chance for a successful transformation. Vector pSW2-RiboA-C9 was successfully transformed into *C. trachomatis* SWFP-, with transformants being obtained in the first passage after transformation under spectinomycin selection. This successful initial transformation supports the notion that previous transformation failures were caused by basal expression of transposase in the un-induced state, as the addition of a riboswitch was the only modification to this vector. Transformants had a normal inclusion phenotype and fluoresced red under UV light ( Figure 2a). However, upon repeated passage under spectinomycin selection, the infectivity of SWFP-/pSW2-RiboA-C9 decreased with each passage until its extinction by passage 4, whilst the control infection (SWFP-/p2TK2-SW2-mCh) expanded normally. We hypothesised that despite improved regulation of expression, the high activity of the C9 variant of the *Himar1* transposase was resulting in low-level basal expression of the enzyme having a disproportionately negative effect on early transformants. Thus, we aimed to reduce the activity of the transposase through mutation.

### Introduction of a point mutation in C9 transposase increases the stability of the plasmid and enables its expansion in cell culture

The C9 mutations of *Himar1* (Q358R and E131K) increase the activity of the transposase by 50-fold ^22^. We reasoned that removing the two C9 mutations in a stepwise manner to give the wild-type coding sequence would decrease the activity of the transposase, and result in an increased stability of the transformants. We chose to first revert the Q358R mutation, changing the amino acid back from arginine (hyperactive variant) to glutamine (wild-type); in *E. coli*, this reduces the activity of the transposase enzyme to less than half that of the C9 mutant ^22^. The vector with this mutation (pSW2-RiboA-C9Q) was successfully transformed into *C. trachomatis* SWFP- on the first attempt, and this time the transformant survived serial passage and expanded in cell culture ( Figure 2b). To ensure the system worked in both chlamydial biovars, we also transformed lymphogranuloma venereum *C. trachomatis* L2(25667R) with pSW2-RiboA-C9Q with the same outcome. We also constructed the other single (E131K) and double (Q358R and E131K) mutants and successfully transformed these into SWFP- and L2(25667R) for future optimisation of the protocol. These were lower activity transposases than the single R358Q mutant ^22^, and their easy isolation was consistent with our hypothesis that reduction of the transposase activity would improve plasmid recovery.

### Immunodetection of C9Q transposase

**Figure 3.**
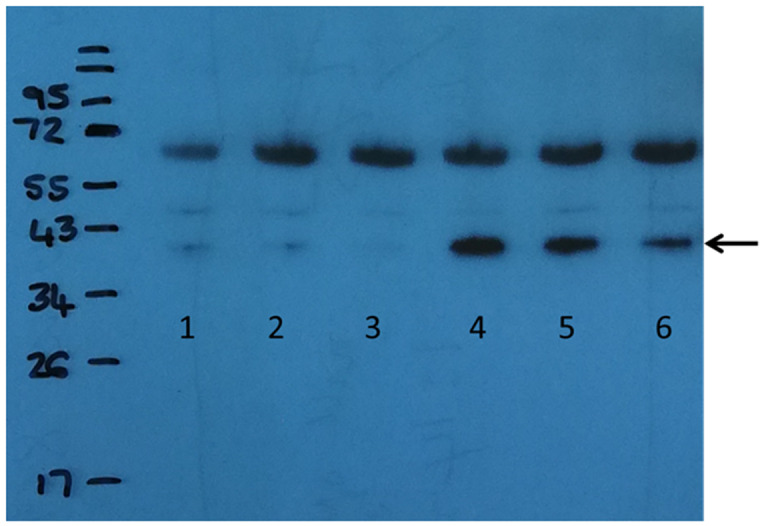
Immunodetection of the C9Q transposase using primary mouse polyclonal antisera to C9 transposase. Lanes 1-3: no ATc induction. Lanes 4-6: induction with 20ng/ml ATc. Lanes 1 and 4: 0mM theophylline; Lanes 2 and 5: 2mM theophylline; Lanes 3 and 6: 10mM theophylline. The transposase protein is indicated by an arrow at about 38 KDa. Additional proteins detected at around 70KDa are due to non-specific binding.

Once a high infectivity preparation was obtained for SWFP-/pSW2-RiboA-C9Q, the next step was to find the optimal concentration of the riboswitch inducer theophylline for use in chlamydia-infected cells. To do this, SWPF-/pSW2-RiboA-C9Q was induced at 24 hours post-infection, with different concentrations of theophylline. After 24 hours further incubation, the infected cells were harvested and subjected to immunodetection with C9 transposase antisera ( Figure 3). It was found that whilst significant induction was seen upon addition of ATc ( *tet* inducer) in comparison with the uninduced controls, the addition of theophylline did not appear to increase this expression; paradoxically, increased theophylline concentration appeared to slightly reduce the amount of expression.

### *C. trachomatis* SWFP- and L2(25667R) transposon mutants were identified by MinION long-read sequencing: proof of principle that inducible transposition from a single self-replicating vector is possible

Transposase expression in *C. trachomatis* strains SWFP- and L2(25667R) was induced at 24 hours’ post-infection. This time point was selected as the standard condition to give enough time for expansion of potential transposon mutants after induction and thus aid in their detection. ATc was added at a concentration previously used for *Chlamydia* (2ngml ^–1^) ^37^ and theophylline was added at 2mM, a standard concentration used in other bacteria ^31^. *Chlamydia* were incubated for a further 18–24 hours after induction to allow completion of the developmental cycle, after which DNA extraction was performed. 400ng of this DNA (including both chlamydial and host cell DNA) was sequenced using the Oxford Nanopore Technologies MinION sequencing device.

**Figure 4.**
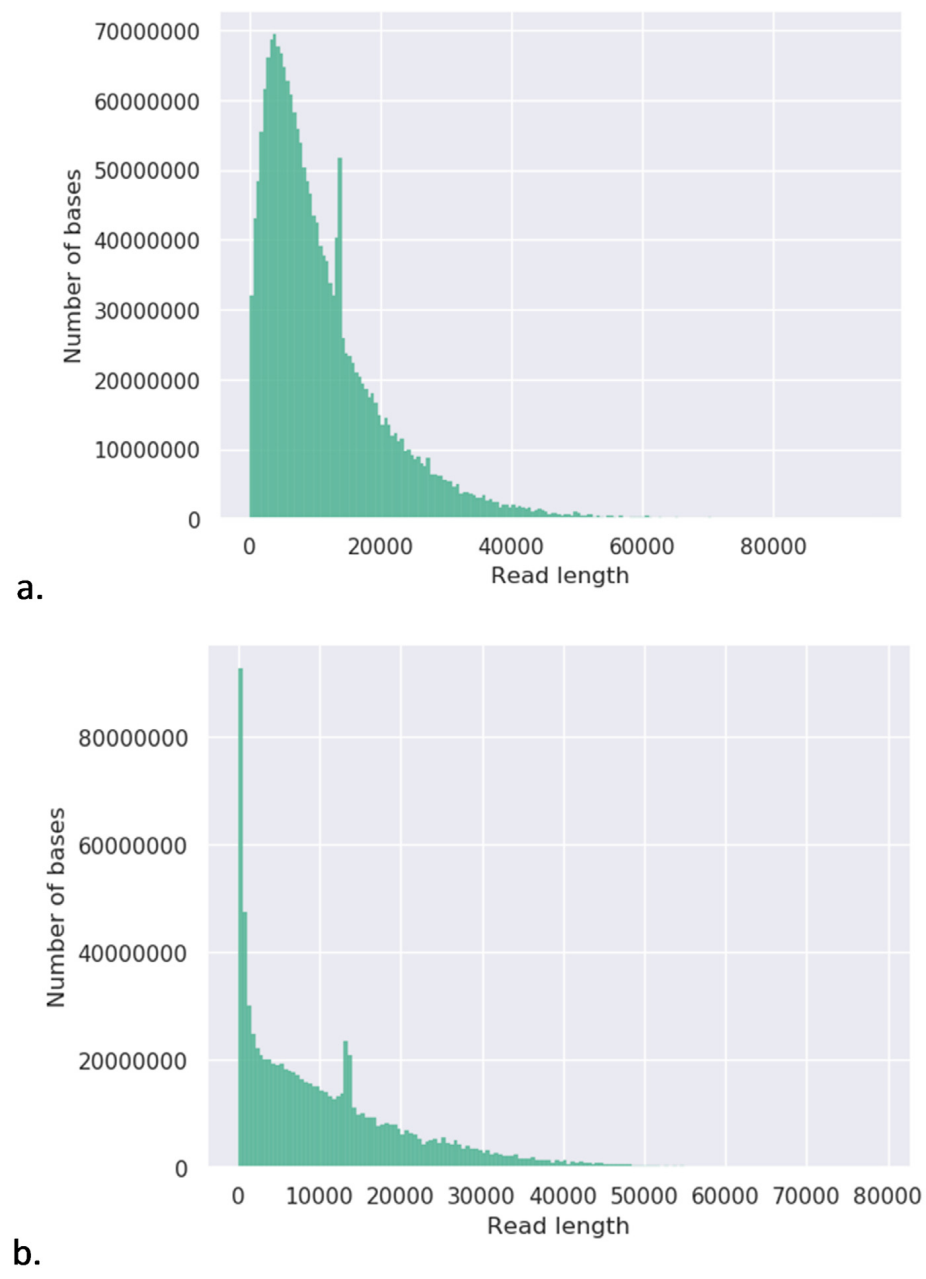
Weighted histograms of read lengths. Histograms were generated from fastq files generated by MinION sequencing, using the Nanoplot (v.1.28.2) ^38^ tool available on the online bioinformatics platform Galaxy. a. *C. trachomatis* SWFP- and b. L2(25667R).

***Transposon insertions identified in* *C. trachomatis* *SWFP- genome*.** In total, there were 468,974 passed reads, all of which had a quality score of >Q7 and were thus included in the analyses. Whilst the longest read was 95,007bp (Q=7.8), the mean read length of passed reads (Q>7) was 4,193bp and the N50 was 8,519 bp. These values may have been slightly skewed by the presence of an obvious sharp, second peak of read lengths between 10–20Kb ( Figure 4a). Given that the delivery vector is 13.8Kb in length, it seems likely that this peak is caused by multiple copies of the vector per chromosome leading to an over-representation of reads of that length.

**Figure 5.**
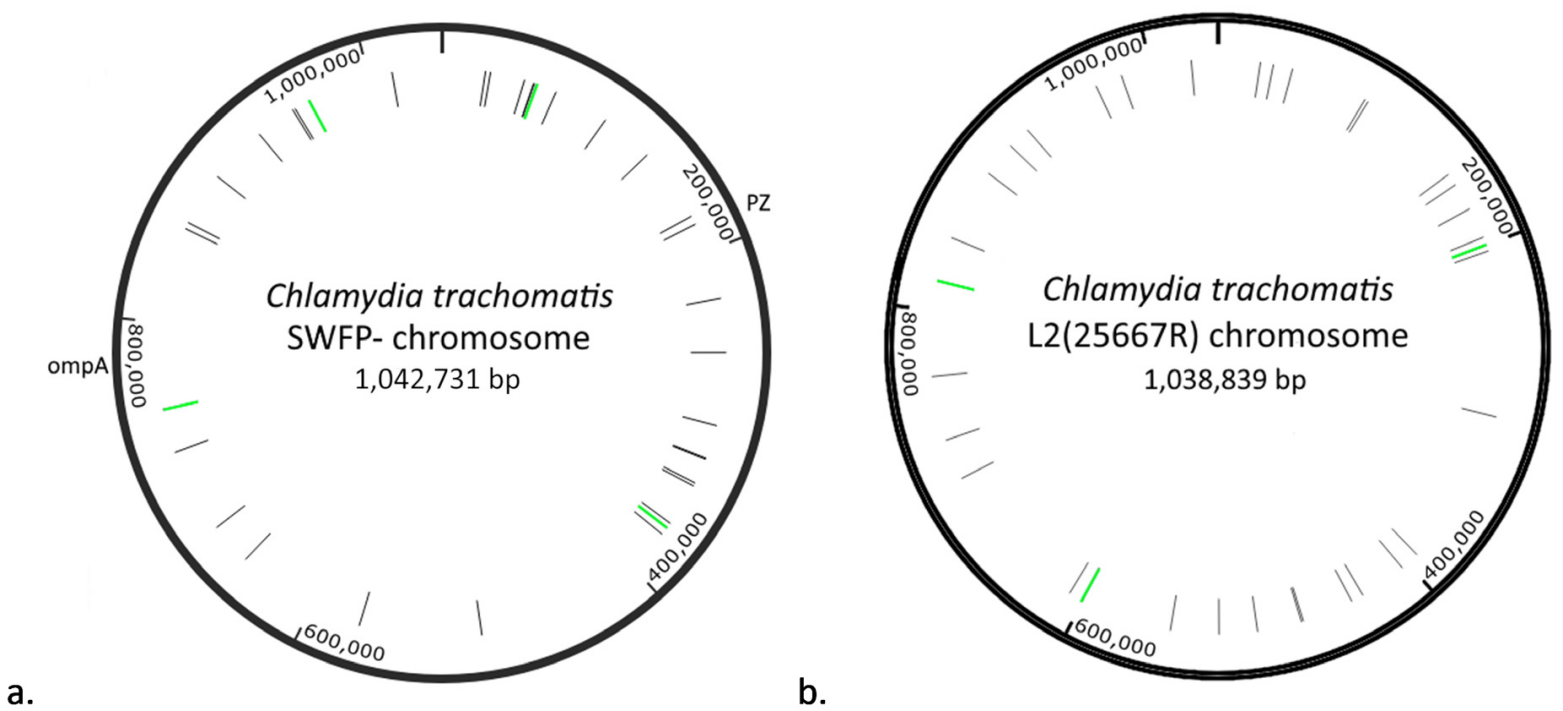
Maps of *C. trachomatis* chromosomes with transposon insert sites indicated. **a**) *C. trachomatis* SWFP- chromosome and **b**) *C. trachomatis* L2(25667R) chromosome. Transposon insertions are indicated in black (single reads of an insertion) or green (multiple reads). The plasticity zone (PZ) and *ompA* gene are indicated for purposes of orientation.

In total, 9188 reads were found to contain the transposon sequence; of these, 43 reads also contained chromosomal sequence from SWFP- (0.46%), suggesting the transposon had inserted in the chromosome. Whilst most reads identified a different insertion, some reads mapped to the same genomic locus and in total 36 unique transposon insertion loci were identified ( Figure 5a and Table 3 ^39^). The two inserts that were identified by more than one read were located in a conserved hypothetical protein (SWFP_0541) at 57,103 bp (6 reads) and in a putative exported lipoprotein SWFP_7041 at 749,933 bp (2 reads). The majority of chromosomal inserts were intragenic, with only three occurring outside of coding sequences. Insertions occurred throughout the genome, although there were some higher- and lower-density areas apparent; most genes contained a single transposon insertion, but some genes had multiple insertions, i.e. there were three insertions into SWFP_0531 which encodes a conserved hypothetical protein, and two insertions in both SWFP_3071 (a candidate inclusion membrane protein) and Polymorphic membrane protein D (pmpD) ( Table 3). 

***Transposon insertions in *C. trachomatis* L2(25667R)*.** In total 472,000 reads were generated from the L2(25667R) transposon mutagenesis library, all of which had a Q>7 quality score. There were 868,400,931 bases among these reads, representing 836 x coverage of the L2(25667R) genome. The read length N50 was 8,381, the longest read was 78,893 bases long (Q=10.3) and the mean read length was 1,839. As seen in SWFP-, a secondary peak is visible between 10–20Kb read length – this is most likely due to presence of the 13.8 Kb transposon delivery vector.

In total, 8,527 reads matched the transposon sequence; of these, 37 reads were identified that mapped to the L2 genome (0.43%). Whilst in most cases the reads identified unique insertion sites in the L2 chromosome, in three cases there were two reads that identified the same insertion site. These were in the intergenic locus 200,325 bp, in the acyl-CoA thioesterase gene (L225667R_RS02850) at 600,083 bp and in the Phosphoenolpyruvate carboxykinase gene (L225667R_RS03810) at 817,240 bp. Thus, in total 34 unique insertion sites were identified ( Figure 5b and Table 3). The distribution of transposon insertions was superficially similar to that seen in the SWFP- chromosome ( Figure 5a), although none of the inserts occurred at the same loci. Only one gene was identified that contained more than one transposon insertion ( Table 3) – two inserts were located in L225667R_RS02165, a predicted Pmp family polymorphic membrane protein autotransporter adhesin.

### Passage of the mutant pool enabled expansion of mutants with permissive gene disruptions

To determine which genes are essential to survival, it is necessary to passage the mutant pool (the input library) so that chlamydia with insertions in essential genes will be lost from the pool and those with permissive insertions will expand. This enables differentiation of essential versus nonessential genes. Whilst the depth of sequencing per mutant is not great enough to identify saturation mutagenesis of the genome using the methods described, in lieu of high throughput sequencing this exercise was intended to identify mutants that survive passage, the first step in differentiating essential from non-essential genes.

SWFP/pSW2-RiboA-C9Q was induced at 24 hours post-infection and grown for a further 24 hours. A quarter of the harvest was then passaged into a fresh monolayer of McCoy cells and grown for 48 hours (forming the output library). At the end of the developmental cycle, 100µl of the input and output libraries were extracted for sequencing by MinION.

***Input library*.** All of the 784,000 reads generated from the input library surpassed the minimum required quality score (Q>7) and were thus included in these analyses ( Table 2). The read length N50 was 1,857 bp, so most of the reads exceeded the length of the transposon sequence (1,230 bp). In total, 4,070 reads were found to contain the transposon sequence, among which 24 reads also matched to a genomic locus (0.59%). Fifteen unique insertion sites were identified ( Table 4). Only two of these sites were intergenic (at 138,399 bp and 468,562 bp), all others fell within coding sequences. All of the insertion events were represented by a single read, apart from one at 57,103 bp which was located in the hypothetical protein gene SWFP_0541, identified by 10 reads. Two other insertions were identified in this gene, each in one read.

**Table 2.** Summary of MinIon sequencing data. This data was generated by Nanoplot (v.1.28.2) using the free online bioinformatics platform, Galaxy (usegalaxy.eu).

Library	Total bases sequenced	Total reads	Mean read length	N50	Number of reads >Q7	% Reads >Q7	Mb reads >Q7
**SWFP- Proof of Principle**	1,966,579,302	468,974	4,193.40	8,519	468974	100	1966.60
**L2(25667R) Proof of Principle**	868,400,931	472,000	1,839.80	8,381	472000	100	868.40
**SWFP- Input library (P1)**	815,057,683	784,000	1,039.6	1,857	784,000	100	815.10
**SWFP- Output library (P2)**	815,472,068	528,000	1,544.50	2,799	501506	95	779.30

**Table 3.** Chromosomal transposon insertion sites in proof of concept experiments for C. trachomatis SWFP- and L2(25667R). Each transposon insertion is listed here, including the genomic location of insert and number of reads identified for that insert, shown in parenthesis. Gene names and predicted protein functions were obtained from the SWFP- and L2(25667R) whole genome annotations (ENA accession numbers HE605380 and HE601954, respectively). Additional information about individual reads can be obtained from the OSF entry for this table.

Strain	Number of Chromosomal insertions	Interrupted gene	Genomic locus of insert (reads)	Gene function
**SWFP-**	36	lepB	25,745 (1)	Signal peptidase I
		prfA	28,831 (1)	Peptide chain release factor 1
		SWFP_0471	48,975 (1)	Putative type III secretion system chaperone
		SWFP_0531	54,985 (1), 55,176 (1), 56,150 (1)	Conserved hypothetical protein
		SWFP_0541	57,103 (6)	Conserved hypothetical protein
		SWFP_0621	68,990 (1)	Putative membrane protein
		scc1	102,255 (1)	Type III secretion chaperone
		SWFP_1201	134,059 (1)	Conserved hypothetical protein
		SWFP_1631	177,698 (1)	MAC/perforin family protein
		SWFP_1671	182,893 (1)	Phosphatidylcholine-hydrolyzing phospholipaseD(PLD) protein (pseudogene)
		SWFP_2131	228,579 (1)	Conserved hypothetical protein
		incB	260,241 (1)	Inclusion membrane protein B
		Pbp	303,236 (1)	Penicillin binding protein
		SWFP_3071	323,110 (1), 324,096 (1)	Candidate inclusion membrane protein
		valS	338,848 (1)	Valine tRNA ligase
		SWFP_3221	340,764 (1)	Conserved hypothetical protein
		SWFP_3481	366,404 (1)	conserved hypothetical protein (pseudogene)
		Intergenic	370,002 (1)	N/A
		dxs	374,308 (1)	1-deoxy-D-xylulose-5-phosphate synthase
		Intergenic	497,622 (1)	N/A
		polA	570,208 (1)	DNA polymerase I
		mutL	647,812 (1)	DNA mismatch repair protein
		sdhA	672,430 (1)	Succinate dehydrogenase
		nqrA	722,727 (1)	Na(+)-translocating NADH-quinone reductase subunit A
		SWFP_7041	748,933 (2)	Putative exported lipoprotein
		23S rRNA	857,032 (1)	23S large subunit ribosomal RNA
		dmpP	860,556 (1)	Na(+)-translocating NADH-quinone reductase subunit F
		murD	892,255 (1)	UDP-N-acetylmuramoylalanine--D-glutamate ligase
		mutS	927,006 (1)	DNA mismatch repair protein
		pmpD	951,586 (1), 952,748 (1)	Polymorphic membrane protein D
		Intergenic	962,605 (1)	N/A
		copD2	1,013,560 (1)	Uncharacterised protein
**L2 (25667R)**	34	L225667R_RS00100	24,794 (1)	isoleucine--tRNA ligase
		trmD	32,481 (1)	tRNA (Guanine-N(1)-)-methyltransferase
		L225667R_RS00200	43,636 (1)	Membrane protein
		recF	88,862 (1)	DNA replication and repair protein
		smpB	91,391 (1)	SsrA binding protein
		L225667R_RS00700	154,091 (1)	Hydrolase
		L225667R_RS00745	161,246 (1)	Hypothetical protein
		L225667R_RS00795	176,896 (1)	MAC/perforin family protein
		L225667R_RS00900	195,362 (1)	Disulfide bond formation protein B
		Intergenic	200,325 (2)	N/A
		L225667R_RS00955	203,457 (1)	Glucose-6-phosphate dehydrogenase
		L225667R_RS01405	300,364 (1)	Penicillin-binding protein 2
		L225667R_RS01820	393,199 (1)	Ribonuclease Z
		L225667R_RS01870	404,455 (1)	Thioredoxin domain-containing protein
		artJ	432,000 (1)	Probable ABC transporter arginine-binding protein ArtJ
		L225667R_RS02040	439,243 (1)	LOG family protein
		L225667R_RS02165	469,083 (1), 470,474 (1)	Pmp family polymorphic membrane proteinautotransporter adhesin
		L225667R_RS02270	496,320 (1)	UPF0158 family protein
		secD	518,570 (1)	SecD/SecF fusion protein
		L225667R_RS02530	546,666 (1)	phenylalanine--tRNA ligase subunit beta
		L225667R_RS02850	600,083 (2)	acyl-CoA thioesterase
		L225667R_RS02900	609,044 (1)	MFS transporter
		L225667R_RS03310	700,842 (1)	Hypothetical protein
		L225667R_RS03415	724,270 (1)	Rod shape-determining protein MreC
		Intergenic	761,933 (1)	N/A
		L225667R_RS03810	817,240 (2)	Phosphoenolpyruvate carboxykinase (GTP)
		L225667R_RS03925	843,195 (1)	Hypothetical protein
		L225667R_RS04090	886,327 (1)	UDP-N-acetylmuramoyl-tripeptide--D-alanyl-D-alanine ligase
		L225667R_RS04185	906,134 (1)	1-acyl-sn-glycerol-3-phosphate acyltransferase
		L225667R_RS04255	919,907 (1)	Hypothetical protein
		L225667R_RS04455	966,486 (1)	Insulinase family protein
		L225667R_RS04525	982,510 (1)	Hypothetical protein
		L225667R_RS04710	1,023,666 (1)	Autotransporter domain-containing protein

**Table 4.** Chromosomal transposon insertion sites in SWFP- from passaging experiment. Input library contains the reads from the induced culture. The output library contains the reads from the passaged input library. Reads show in bold typeface are those which were identified both in the input and output libraries. Additional information about the reads can be found in the OSF entry for this table.

Library	Number of Chromosomal insertions	Gene name	Genomic locus of insert	Gene function
**Input** **library**	15	SWFP_0531	55,168 (1)	Conserved hypothetical protein
		SWFP_0541	57,006 (1), **57,103 (10),** **57,210 (1)**	Conserved hypothetical protein
		SWFP_0551	**58,784 (1)**	Conserved hypothetical protein
		Intergenic	138,399 (1)	N/A
		SWFP_1441	155,576 (1)	Microsomal dipeptidase
		SWFP_1661	182,100 (1)	Phosphatidylcholine-hydrolyzing phospholipase D(PLD) protein
		SWFP_1731	188,959 (1)	Putative cytoadherence factor (fragment)
		SWFP_2051	221,318 (1)	Putative integral membrane protein
		SWFP_3021	315,268 (1)	Conserved hypothetical protein
		clpC	318,771 (1)	Probable ATP-dependent Clp protease ATP-binding subunit
		SWFP_3481	367,078 (1)	Conserved hypothetical protein (pseudogene)
		Intergenic	468,562 (1)	N/A
		parB	791,306 (1)	Probable chromosome-partitioning protein ParB
**Output library**	33	SWFP_0531	55,176 (4), 55,658 (1), 55,698 (1)	Conserved hypothetical protein
		SWFP_0541	**57,103 (47),** **57,210 (1),** 57,330 (1)	Conserved hypothetical protein
		SWFP_0551	58,456 (1), 58,729 (3), **58,784 (1),** 59,519 (1)	Conserved hypothetical protein
		Intergenic	72,564 (1)	N/A
		SWFP_1381	150,928 (1)	Conserved hypothetical protein
		SWFP_1661	182,033 (1)	Phosphatidylcholine-hydrolyzing phospholipase D(PLD) protein
		tgt	217,967 (1)	Queuine tRNA-ribosyltransferase
		hemL	239,202 (1)	Glutamate-1-semialdehyde 2,1-aminomutase
		Intergenic	255,327 (1)	N/A
		aroE	423,674 (1)	Shikimate biosynthesis protein AroDE
		pmpB	476,039 (1), 476,410 (1)	Polymorphic membrane protein B
		euo	517,287 (1)	Early upstream open reading frame
		uvrB	662,254 (1)	UvrABC system protein B
		SWFP_6361	667,483 (1)	Putative exported protein
		rpoN	691,087 (1)	RNA Polymerase Sigma-54
		SWFP_6831	719,973 (1)	Conserved hypothetical protein
		SWFP_7041	749,048 (1)	Putative exported lipoprotein
		rrf	776,333 (1)	Ribosome-recycling factor
		porB	826,298 (1)	Outer membrane protein B
		SWFP_7931	840,853 (1), 841,236 (1)	Putative integral membrane protein
		SWFP_8391	898,251 (1)	Conserved hypothetical protein
		SWFP_8401	898,999 (1)	Putative membrane protein
		SWFP_9321	998,483 (4)	Conserved hypothetical protein
		pmpG	1,034,840 (1)	Polymorphic membrane protein G

***Output library*.** In the output library, there were 528,000 reads, 501,506 of which had a quality score of Q>7 ( Table 2). The read length N50 was 2,799bp. In total, 4,511 reads contained the transposon sequence, 87 of which were found to also match a genomic locus (1.92%); of these, 33 unique insertion sites were identified ( Table 4). Two of these sites were intergenic (72,564 bp and 255,327 bp – each present in a single read); all others fell within coding sequences. Four of the transposon insertion sites were identified by multiple reads: an insert in gene SWFP_0531 was present in four reads (two further inserts in this gene were identified in single reads); the insert in SWFP_0541 (at position 57,103bp) was identified in 47 reads (two further inserts identified in this gene in single reads); an insert in SWFP_0551 was present in three reads (three additional inserts in this gene were identified in single reads); the insert in SWFP_9321 was present in four reads. The remaining 29 transposon insertions were each identified in a single read.

***Comparison of input and output libraries*.** Of the 15 unique insertions identified in the input library, 3 were also identified in the output library: the commonly identified insert in SWFP_0541 (at 57,103 bp), which was identified in 10 reads in the input library and 47 reads in the output library; a second insert site in SWFP_0541 (at 57,210 bp) which was identified in a single read in both input and output libraries and SWFP_0551 (at 58,784 bp), which was again present in a single read in both the input and output libraries. None of the other mutations were identified in the output library, suggesting they were either deleterious and therefore did not survive passage, or were below the limit of detection in these experiments.

Additional gene disruptions were identified in the output library that were absent from the input library (also see Table 4): SWFP_0531 contained three separate insert sites, one at 55,176bp, present in four reads, one at 55,658 bp (one read) and one at 55,698 bp (one read); SWFP_0551 (at 58,456 bp, present in one read; 58,729bp, present in three reads and 59,519 bp, present in one read) and SWFP_9321 (at 998,483bp, present in four reads). The other gene disruptions present only in the output library (all identified by single reads) were: SWFP_0541, SWFP_1381, SWFP_1661, *tgt*, *hemL*, *aroE*, *pmpB* (2 inserts identified), *euo*, *uvrB*, SWFP_6361, *rpoN*, SWFP_6831, SWFP_7041, *rrf*, *porB*, SWFP_7931 (2 inserts identified), SWFP_8391, SWFP_8401 and *pmpG*.

## Discussion

Transposon mutagenesis is a widely used tool for gene function analysis and has been applied to diverse species, both prokaryotic and eukaryotic. However, until now only two studies have successfully applied this methodology to the *Chlamydiaceae* ^20, 21^. Both studies used a non-replicating transposon delivery vector, with the transposase expressing from a constitutive chlamydial promoter. This resulted in very low numbers of transposon insertions per experiment, reflecting the low transformation efficiency of *Chlamydia*. In the present study we have addressed this limitation by placing all of the necessary transposon mutagenesis components onto a single, self-replicating vector so that transposition could be induced once a high infectivity is reached.

Early iterations of the transposon delivery vector failed to transform *C. trachomatis*, whilst vectors carrying either the transposon or transposase in isolation transformed with ease ^30^. This suggested that basal expression of transposase from the *tet* promoter was causing premature transposition and killing of early transformants – either due to transposition into essential genes or loss of the vector after transposition. It has been shown that upon excision of the transposon by transposase, the resulting double-stranded break is either repaired through homologous recombination (if a template is available) or the vector is degraded (“donor suicide”) ^40, 41^. If only one vector is present, (which is the most likely scenario if transposition occurs prior to replication in an early transformant), there will be no template to copy and thus the vector will be lost. Whilst the transposon is likely to “jump” into the chromosome, conferring beta lactam resistance to the recipient, this single chromosomally located copy may not allow sufficient protection of the developing chlamydium upon penicillin selection. 

Replacement of the *tet* promoter in the transposase regulon seemed unlikely to solve the problem of premature transposition as basal expression is an evolutionary advantage that enables a fast response to rapidly changing environments and is therefore a feature of all naturally-occurring bacterial promoters ^42^. Therefore, as an alternative solution, we added a secondary control element to the regulon in the form of a riboswitch to reduce the impact of prematurely transcribed mRNA by preventing its translation.

Riboswitches are comprised of short DNA sequences in the 5' untranslated regions of a gene, downstream of the promoter, which make up the aptamer (substrate-binding) domain and expression platform (usually an intrinsic transcription terminator ^43^); these interact to form a stem and loop structure when the mRNA is transcribed ^43, 44^. This structure is highly sensitive to a specific substrate and in nature often forms an integral part of the expression control unit of biosynthetic operons ^45^. Once the aptamer binds to its substrate, the confirmation of the riboswitch changes so that it is either activated or inactivated, depending on the function of the riboswitch (i.e. on in the presence of the substrate, or off in the presence of the substrate). Riboswitches were first engineered fifteen years ago, with the advent of the theophylline-sensing riboswitch for use in detection of small molecules by *Escherichia coli* ^46^. The theophylline riboswitch has since been adapted for use in multiple species (both prokaryotic and eukaryotic) including *Agrobacterium tumefaciens*, *Acinetobacter baylyi*, *Acinetobacter baumannii*, *Mycobacteria smegmatis*, *Bacillus subtilis* and *Streptococcus pyogenes* ^31^, *Streptomyces coelicolor* ^47^, *Leishmania major* ^48^, *Synechoscystis sp*. ^49^ and *Synechococcus elongates* ^50^. Naturally-occurring riboswitches have been documented to exist in *C. trachomatis* ^51^, and in combination with the wide host-range suitability of the theophylline riboswitch, this suggested its potential utility in *C. trachomatis.*

A number of theophylline riboswitches have been engineered, each with varying levels of expression in the activated or inactivated state, depending on the species of bacteria used ^31^. Riboswitch A was selected as a starting point, as basal expression from a constitutive promoter incorporating this riboswitch was very low in most species tested. The expression in the presence of theophylline was also found to be very low in most cases, but we reasoned that the hyperactivity of the C9 transposase would likely compensate for its low-level expression.

The first attempt to transform *C. trachomatis* strain SWFP- with pSW2-RiboA-C9 was a success, with red fluorescent, spectinomycin-resistant inclusions identified at the first passage after transformation. This supported the notion that basal expression accounted for the previous transformation failures ^30^, as the addition of a riboswitch was the only modification to the vector. However, whilst the addition of a riboswitch enabled initial successful transformation of SWFP- with the transposon delivery vector, upon repeated passage the transformant did not expand as expected; rather, its infectivity decreased with each subsequent passage and was lost at passage 6. Repeated transformation resulted in the same outcome. This led to the conclusion that despite tighter control of transposase expression, some transposition was still occurring, killing early transformants. It seemed reasonable that a tiny amount of un-induced expression may still have an effect due to the hyperactivity of the C9 transposase, so to counteract this, the activity of the transposase enzyme was reduced through site-specific mutation. To do this, each of the two mutations that confer hyperactivity, both in succession and in combination, were returned to their wild-type sequences (R131Q and K137E). All three mutated plasmids successfully transformed *C. trachomatis* SWFP-, and survived serial passage in cell culture, confirming our hypothesis that the hyperactivity of C9 was responsible for the apparent low fitness of transformants. The single R131Q mutant is the most active of the three transposase mutants, 20-times more active than the wild-type himar1 transposase but less than half as active as the C9 mutant in *E. coli* ^22^. SWFP- and L2(25667R) transformed with the shuttle vector carrying this version of the transposase gene, (pSW2-RiboA-C9Q), were induced mid-way through the developmental cycle with the hope that resulting transposon mutants would expand, making them easier to detect. For gene function analysis, the mutant library would be passaged at the end of the developmental cycle to recover the surviving population; however, in these initial experiments, sequencing was performed directly on this mutant “input” library to obtain proof of transposition.

TraDIS data is usually generated using Illumina-based, short-read sequencing platforms such as the HiSeq or MiSeq and a convenient bioinformatics pipeline in the form of the TraDIS Toolkit is available for the semi-automated identification of transposon insertion sites from such datasets ^34^. However, it was not possible to access commercial sequencing facilities during the coronavirus pandemic, so the transposon mutant libraries generated in this study were sequenced using a locally available MinION nanopore sequencing device. This is not without precedence: one other report has used the MinION to identify transposon mutants to date, which proved useful in devising a bioinformatics pipeline appropriate for identifying transposon insertions in long-read sequencing data ^36^. The read-length produced by nanopore sequencing typically exceeds that of the transposon sequence, allowing for easy identification of the genetic context of inserted transposons – in the proof of principle experiments presented here, the mean read-length was 1,839 bp for L2(25667R) and 4,193 bp for SWFP-, which are both sufficient to encompass the whole inserted transposon and flanking sequences to easily locate the site of insertion (although this is not an absolute requirement and reads frequently contained only part of the transposon and one flanking sequence). However, there are two drawbacks of using the MinION rather than high-throughput, short-read sequencing platforms. Firstly, whilst the technology is continually improving, the base-calling accuracy is still relatively low ^52– 54^. Whilst this may pose a problem in *de novo* genome sequencing, the identification of a transposon insertion site relies upon alignment of transposon-containing reads to a known reference genome so provided enough of the chlamydial flanking sequence is present the read will still map to the correct location irrespective of the presence of errors. Secondly, the depth of sequencing achievable by MinION is far less than that produced by a high-throughput, short-read sequencer ^55^. This limitation can be overcome by running multiple flow-cells in parallel, a capability offered by the high-throughput GridION and PromethION platforms – but as the intention of this study was the development of an inducible transposon mutagenesis system for *Chlamydia* rather than saturation mutagenesis, the MinION was sufficient to provide proof-of-principle and for similar studies offers a cost- and time-effective alternative to short-read sequencing.

Given that the DNA analysed in this study was obtained directly from a harvested *Chlamydia* culture and not purified elementary bodies (EBs), much of the DNA present would have been from the host cells rather than *Chlamydia*. EB purification was avoided to keep the protocol as simple as possible but may have resulted in an underrepresentation of transposon-containing reads. Nevertheless, the methods employed here enabled the identification of 36 and 34 unique transposon insertion sites in the first sequencing attempts of SWFP- and L2(25667R), respectively, in the first un-optimised experiments, proving that inducible transposon mutagenesis from a single replicating vector is possible and results in multiple transposition events in *C. trachomatis*.

Most transposon inserts were located within coding sequences of the chromosome, consistent with the high protein-coding density of the *C. trachomatis* genome ^1^. We did not attempt to identify insertions in the plasmid as naturally-occurring plasmid-free *Chlamydia* exist and thus it is implicit that plasmid genes are not essential to survival in cell culture; furthermore, as there are only eight coding sequences in the plasmid, targeted mutagenesis is sufficient for gene function analysis. Transposon insertion sites were well distributed around the chromosome, but some clustering was observed, with some genes containing more than one unique insertion site ( Table 3). Indeed, one intergenic insertion site (at 57,103 bp) occurred in both the SWFP- proof-of-principle experiment and in the passaging experiment. Although *Himar1* transposons randomly insert at TA dinucleotides ^56^, they are also known to have a preference for bent or bendable DNA ^57^, and in closely related transposon *Hsmar1*, DNA topology of both the donor and recipient molecule was found to be important ^58^. This may explain the clustering seen in the present study and is an import consideration for saturation mutagenesis experimental design, as it may confound the identification of essential genes ^59^.

Saturation mutagenesis is defined as the point at which the discovery of new transposon insertions plateaus and thus all possible insertion sites have been identified ^59^. Assuming insertion is unbiased, for *C. trachomatis* SWFP- this would mean 68,728 insertions, i.e. the number of TA sites present in the chromosome. The aim of saturation mutagenesis is to enable easy differentiation of essential and non-essential genes – this is because it is possible for a gene to be mutated without affecting the protein function, for example if the insertion occurs towards the 3’ end of the coding sequence, so multiple insertions in a gene are needed at different sites to allow for differentiation. This is possible with the system presented here, providing that 1. a high enough titre of *C. trachomatis* is present at the time of transposition, which is enabled by the tightly controlled regulon developed in this study; 2. a balance between transformability and transposase activity can be reached (i.e. through further modifications to the riboswitch or transposase gene) and 3. depth of sequencing is sufficient to identify all transposon insertions present. The latter can be optimised by increasing the amount of input DNA processed for library preparation, the relative amount of chlamydial DNA present (EB purification may be required; alternatively an amplification-based sequencing method could be used to increase the proportion of DNA containing transposon-chlamydia genomic DNA junctions in the libraries) and/or changing the method used for sequencing to allow a greater depth of coverage. If saturation mutagenesis is not possible in a single experiment due to biological or technical constraints, it is possible to pool the data from multiple transposon mutant libraries to achieve the required number of mutations ^59^.

This study provides proof-of-principle that transposon mutagenesis can be controlled in *C. trachomatis*. Whilst we did not attempt to prove saturation mutagenesis in this study, an experiment towards this next step was performed to investigate whether passage of an induced pool of chlamydia would result in the expansion of permissive mutants (i.e. *Chlamydia* with inserts in non-essential genes). Indeed, expansion of the mutation in SWFP_0541 (at 57,103 bp) suggests that this gene is nonessential, given that 10 reads identified this mutation in the input library (prior to passage) – further supported by its identification in the proof of principle experiment for SWFP-. This also suggests that induction of the transposase and insertional mutagenesis occurred prior to replication of that mutant, as intended by inducing expression of transposase mid-way through the developmental cycle. This mutation in SWFP_0541 survived passage, with 47 reads containing the transposon sequence at 57,103 bp being identified in the output library. Two other mutations identified in the input library were found in the output library, but these were only identified by single reads, suggesting a low abundance of these mutants in the pool. The rarity of these reads may suggest that they resulted from carry-over from the input library rather than an active infection by these mutants. It is also possible that these mutants underwent limited rounds of replication following induction, but had lost their infectivity and hence did not survive passage; or that infectivity was maintained but replication was limited by the mutation. The depth of sequencing per mutant is too low to examine this (i.e. in most cases only a single representative read identified each mutant), but the identification of mutants with interruptions in known essential genes within the output library (e.g. *uvrB* and *euo*) certainly supports carry-over of mutated DNA ( Table 4). Whilst this is a limitation of the methods used, the recent re-opening of high-throughput sequencing facilities will now enable the statistical differentiation of carry-over DNA from true survival of mutants.

Whilst other studies have also used riboswitches as part of a dual control regulon (as recently reviewed by Kato ^60^), they were initially developed for use with a constitutively expressing promoter as the sole regulatory element so can be expected to offer adequate expression control in their own right ^46, 61^. This is significant as currently there is only one inducible expression system available in Chlamydia ^37^. In the present study, whilst the addition of a riboswitch to the regulon evidently stabilized the plasmid through reducing the effect of uninduced transcription, we were surprised the addition of theophylline did not result in an increase in protein expression ( Figure 3). This may be explained by the low responsiveness of riboswitch A to theophylline, as previously described ^31^ – it seems the presence of the riboswitch reduces translation when only a small amount of mRNA is present (i.e. in the uninduced state), but addition of the theophylline substrate only has a modest effect on translation in the induced state. It could also be that theophylline is unable to access the replicating RBs within the chlamydial inclusion. However, in experiments developing theophylline-induced riboswitches in Mycobacteria, theophylline induction was achieved in cell cultures of *Mycobacterium tuberculosis* ^62^. Given that *M. tuberculosis* occupies a host-derived vacuole within human macrophages (the phagosome), a similar mechanism of intracellular survival to *C. trachomatis*, it seems likely that intra-inclusion induction by theophylline is possible in the Chlamydiae. However, further experiments utilizing reporter gene assays are needed to investigate theophylline activation of riboswitches in *C. trachomatis*.

An alternative hypothesis that may explain our findings is offered by recent evidence obtained by Ouellette *et al.* (2021) who found that introducing a weak ribosome binding site overcomes leaky protein expression from the *tet* promoter ^63^. Further testing and optimisation of riboswitches in *Chlamydia* is needed to resolve this, and alternative riboswitch designs (and perhaps a *Chlamydia*-specific riboswitch) may be required if they are to offer an alternative to the *tet* promoter for inducible protein expression in *Chlamydia*.

## Conclusions

In this study we have engineered the first stable self-replicating chlamydial transposon delivery vector that carries a tightly regulated transposase, enabling the fine tuning of transposon mutagenesis experiments. Using the MinION, we were able to identify 36 and 34 individual transposon insertions into the SWFP- and L2(25667R) chlamydial chromosomes in a single induction experiment. This is likely to be a significant underrepresentation of the actual number of insertions and optimisation of the protocols including the use of high throughput sequencing in future experiments will undoubtedly reveal many more. Successful transformation relied upon the incorporation of a riboswitch into the transposase regulon which provides a possible alternative, translation-based method of regulating gene expression in *C. trachomatis*; however, mutation of the C9 transposase was also needed to slightly reduce its activity and ensure stability of the vector upon repeated passage. Together, the methods described in this study pave the way to developing a saturation transposon mutagenesis protocol for gene function analysis in *C. trachomatis*, which could ultimately lead to the identification of novel targets for drug or vaccine development.

This work has wide-ranging implications on the future of *Chlamydia* genetics, as successful transformation of four other chlamydial species has been achieved thus far ^64– 66^ and so the transposon mutagenesis approach we describe here could be easily adapted to these.

## Data availability

### Underlying data

Open Science Framework: An inducible transposon mutagenesis approach for *Chlamydia trachomatis*. https://doi.org/10.17605/OSF.IO/Y9RQG ^39^.

This project contains the following underlying data:

- The annotated sequence of the synthesised Himar1_RiboA_C9 cassette

- The complete annotated sequence of pSW2-RiboA-C9 (FastA file)

- The annotated sequence of the mutated region in pSW2-RiboA-C9Q (FastA file)

- Underlying images for Figures 1, 2, 3 and 5

- Underlying data for tables 3 and 4 (.xlsx) (read info for genome transposon insertions)

In addition, all sequencing reads from this project are available on the Sequence Read Archive (SRA) available at https://www.ncbi.nlm.nih.gov/sra via the BioProject ID PRJNA770870. Read files from individual libraries can be accessed from the following links:

NCBI: Induced C. trachomatis SWFP- (proof-of-principle); https://www.ncbi.nlm.nih.gov/biosample/22242659.

 NCBI: Induced C. trachomatis L2(25667R) (proof-of-principle); https://www.ncbi.nlm.nih.gov/biosample/22242660.

NCBI: Induced C. trachomatis SWFP- (Input library); https://www.ncbi.nlm.nih.gov/biosample/22242661.

NCBI: First passage of induced C. trachomatis SWFP- (Output library); https://www.ncbi.nlm.nih.gov/biosample/22242662.

Data are available under the terms of the Creative Commons Zero "No rights reserved" data waiver (CC0 1.0 Universal Public domain dedication).
