# Supplementary material for: An inducible transposon mutagenesis approach for the intracellular human pathogen Chlamydia trachomatis
Source: Wellcome Open Res. 2021 Nov 16;6:312. [Version 1] doi: 10.12688/wellcomeopenres.16068.1 (PMC8767425; doi:10.12688/wellcomeopenres.16068.1)
Supplement: Peer review history

# Peer review: Reviewer response for version 1

**DOI:** 10.21956/wellcomeopenres.17627.r47092

**Published:** 2022-01-18

**Authors:** Garry Myers

**Affiliations:** 1 The iThree Institute, University of Technology Sydney, Sydney, NSW, Australia

Members of the genus *Chlamydia*, including the human pathogen *C. trachomatis*, have a significant global burden on health in humans and animals. Understanding of these intracellular bacteria, and thus any improved treatments, has been slow, owing to the fact that *Chlamydia* has been mostly recalcitrant to genetic manipulation for many decades. 

 Reliable transformation was only developed in 2011 (led by the senior author of this work). Transposon mutagenesis was subsequently described but only in a non-replicating vector; combined with the poor transformational efficiency of *Chlamydia*, this limited the number of mutants obtainable. In this key work, O'Neill *et al. *describe a stable inducible replicating vector; this was achieved by use of a riboswitch combined with mutagenesis of the hyperactive transposase to further reduce basal expression.

 O'Neill *et al.* then apply this construct to perform careful preliminary TraDIS-style experiments on *in vitro Chlamydia* infections as a proof-of-principle for saturating mutagenesis. This proof-of-principle is successful, identifying 70 transposon insertions, and the data is convincing, despite the impact of the COVID pandemic on sequence data collection. This work represents an important inflection point in chlamydial research.

1. How do the obtained transposon insertion sites in these experiments compare to those previously obtained with non-replicating vectors (despite the low numbers)?
2. What strategies might be effective to overcome potential DNA topology effects in forthcoming saturation experiments?
3. This work is performed *in vitro*. Given that gene essentiality is likely to be substantially different in *in vivo* tissue contexts, is there an expectation that this construct and mutagenesis strategy will be effective in animal models?

Is the work clearly and accurately presented and does it cite the current literature?

Yes

If applicable, is the statistical analysis and its interpretation appropriate?

Yes

Are all the source data underlying the results available to ensure full reproducibility?

Yes

Is the study design appropriate and is the work technically sound?

Yes

Are the conclusions drawn adequately supported by the results?

Yes

Are sufficient details of methods and analysis provided to allow replication by others?

Yes

Reviewer Expertise:

chlamydial biology, microbial genomics, infection biology

I confirm that I have read this submission and believe that I have an appropriate level of expertise to confirm that it is of an acceptable scientific standard.

# Peer review: Reviewer response for version 1

**DOI:** 10.21956/wellcomeopenres.17627.r47094

**Published:** 2021-12-15

**Authors:** Isabelle Derré

**Affiliations:** 1 Department of Microbiology, Immunology, and Cancer Biology, University of Virginia, Charlottesville, VA, USA

*Chlamydia trachomatis* is the leading cause of bacterially associated sexually transmitted infections. The identification of bacterial factors essential for interaction with the host is critical to design interventions to prevent infection. However, this process is complicated by the obligate intracellular life style of the pathogen and the limited genetic tools available to conduct genome wide mutagenesis.

 To address this gap, O’Neill and colleagues have designed an inducible transposon mutagenesis system for *Chlamydia*. The proof of principle experiments presented here demonstrate 1) successful and efficient transposition, leading to multiple transposition events and 2) technical feasibility of assessing infectivity of a pool of mutant.

 Major improvements, compared to the previously reported transposition system for *Chlamydia* are:

1. a self-replicating vector that harbors both the transposon and the transposase; therefore, bypassing low transformation efficacy of non-replicating vectors.
2. dual transcriptional and translational control of a transposase coupled with reduced transposase activity, to mitigate toxicity previously observed due to premature transposition.

Transposition was achieved in two *C. trachomatis* biovars (urogenital and LGV) and resulted in ~35 unique insertions each, in a single non-optimized experiment, which is very promising for an end goal of saturated mutagenesis. Additionally, the majority of transposon insertion sites were intragenic, and for the most part evenly distributed throughout the genome.

 The authors also present a proof-of-concept experiment to assess the infectivity of transposition mutants by sequencing and comparing input (original mutant pool after transposase induction) to output (mutants recovered after one passage of the original mutant pool) mutant libraries. The small sample size does not allow to draw conclusions on potential essential genes; however technical feasibility is clearly established.

 Additional gene disruptions were identified in the output pool, compared to the input pool. This could reflect limitations in the sequencing approach, but I was wondering if this result could also be explained by additional true transposition events occurring during the passage of the input pool and due to low level of expression of the transposase. Did the authors perform sequencing on input “mutant” libraries from uninduced cultures?

 Overall, the manuscript is well written, the results are presented clearly and limitations are discussed. Altogether, the results presented here are very promising and nicely complement previous efforts to develop efficient transposition mutagenesis in *Chlamydia*, bringing the field one step closer to achieving saturated transposition mutagenesis in *Chlamydia* and identifying essential genes of this important obligate intracellular pathogen.

Is the work clearly and accurately presented and does it cite the current literature?

Yes

If applicable, is the statistical analysis and its interpretation appropriate?

Not applicable

Are all the source data underlying the results available to ensure full reproducibility?

Yes

Is the study design appropriate and is the work technically sound?

Yes

Are the conclusions drawn adequately supported by the results?

Yes

Are sufficient details of methods and analysis provided to allow replication by others?

Yes

Reviewer Expertise:

Chlamydia

I confirm that I have read this submission and believe that I have an appropriate level of expertise to confirm that it is of an acceptable scientific standard.
